# The Prognostic Role of Circulating Tumor Cells (CTCs) in Lung Cancer

**DOI:** 10.3389/fonc.2018.00311

**Published:** 2018-08-14

**Authors:** Joanna Kapeleris, Arutha Kulasinghe, Majid E. Warkiani, Ian Vela, Liz Kenny, Kenneth O'Byrne, Chamindie Punyadeera

**Affiliations:** ^1^School of Biomedical Sciences, Institute of Health and Biomedical Innovation, Queensland University of Technology, Kelvin Grove, QLD, Australia; ^2^Translational Research Institute, Brisbane, QLD, Australia; ^3^School of Biomedical Engineering, University of Technology Sydney, Sydney, NSW, Australia; ^4^Institute of Molecular Medicine, I.M. Sechenov First Moscow State Medical University, Moscow, Russia; ^5^Department of Urology, Princess Alexandra Hospital, Woolloongabba, QLD, Australia; ^6^Australian Prostate Cancer Research Centre, Institute of Health and Biomedical Innovation, Translational Research Institute, Queensland University of Technology, Princess Alexandra Hospital, Brisbane, QLD, Australia; ^7^School of Medicine, University of Queensland, Royal Brisbane and Women's Hospital, Central Integrated Regional Cancer Service, Queensland Health, Brisbane, QLD, Australia; ^8^Princess Alexandra Hospital, Queensland Health, Brisbane, QLD, Australia

**Keywords:** lung cancer, NSCLC, SCLC, Circulating tumor cells, liquid biopsy

## Abstract

Lung cancer affects over 1. 8 million people worldwide and is the leading cause of cancer related mortality globally. Currently, diagnosis of lung cancer involves a combination of imaging and invasive biopsies to confirm histopathology. Non-invasive diagnostic techniques under investigation include “liquid biopsies” through a simple blood draw to develop predictive and prognostic biomarkers. A better understanding of circulating tumor cell (CTC) dissemination mechanisms offers promising potential for the development of techniques to assist in the diagnosis of lung cancer. Enumeration and characterization of CTCs has the potential to act as a prognostic biomarker and to identify novel drug targets for a precision medicine approach to lung cancer care. This review will focus on the current status of CTCs and their potential diagnostic and prognostic utility in this setting.

## Introduction

Lung cancer is the leading cause of cancer-related mortality among men and women worldwide (1). In 2012, the incidence was estimated at 1.8 million new cases, accounting for 12.9% of all new cancers diagnosed globally (2). There is an estimated 18% survival rate beyond 5 years for all stages combined, with poor outcomes largely due to late diagnosis (1, 3). The majority of patients present with locally advanced or metastatic disease, with ~20–30% of patients presenting with early stage disease (3, 4). Late diagnosis is a major underlying cause for this advanced disease presentation (5). The annual mortality rate for lung cancer is higher than for colon, breast, and prostate cancers combined (6). The majority of patients presenting with advanced stage at diagnosis contributes to this poor outcome (4).

There are two main types of lung cancers, small cell lung carcinoma (SCLC) and non-small cell carcinoma (NSCLC). NSCLC is the most common, accounting for 80% of cases (7). NSCLC has three main histological subtypes: adenocarcinoma, squamous cell (epidermoid) carcinoma, and large cell undifferentiated carcinoma. Adenocarcinoma accounts for ~40% of cases although is increasing in relative incidence, and usually starts in mucus secreting epithelial cells (167). The prognosis of NSCLC subtypes depends on the stage of the tumor and the treatment availability.

Small cell lung cancer (SCLC) accounts for about 15% of all lung cancers diagnosed annually and up to 25% of lung cancer deaths. SCLC is characterized by a more aggressive clinical phenotype than NSCLC with progression to metastatic disease earlier in the disease course (8).

SCLC and NSCLC arise from different cell types and demonstrate varying clinical features as shown in Table 1.

**Table 1:** Lung cancer classification.

		**Location in the lung**	**Common features**	**Common mutations**
NSCLC (80–85%)	Adenocarcinoma (40%)	Peripheral	Most common type of cancer in non- smokers More common in women Should test for EGFR, ALK, ROS1 and BRAF mutation for targeted therapy	EGFR KRAS CDKN2A ALK BRAF MET TP53
	Squamous cell carcinoma (25-30%)	Central and Peripheral	Strongly associated with cigarette smoking	
	Large cell carcinoma (10-15%)	Peripheral	Similar characteristics to adenocarcinoma	
SCLC (15–20%)		Central	Strongest association with smoking Very rapid growth Early distant metastasis in brain, liver and bone Worst prognosis Better response to chemotherapy	MYC BCL2 c-KIT TP53 RB

Lung cancer may be initiated through exposure to carcinogens. The main risk factor for lung cancer is the use of tobacco. Tobacco is known to initiate and promote carcinogenesis and accounts for 85% of lung cancer cases (9). Additional known risks include exposure to pollutants such as asbestos, tar and metals including arsenic, and chromium. Common symptoms include persistent cough, worsening breathing, pneumonia that fails to resolve, chest discomfort, wheezing, blood in the sputum, and hoarseness (3, 10). A minority are asymptomatic, detected by chance through investigation of other illnesses or in screening programs (11).

Treatment options depend on the intent of treatment and may include loco-regional treatment such as surgery, image guided ablation including radical chemo-radiotherapy, stereotactic ablative radiation treatment, thermal ablation or cryotherapy, or systemic treatment such as chemotherapy, targeted agents, and immunotherapy, alongside novel agents under current investigation in clinical trials (11). An example of the power of targeted therapies in a precision medicine approach was demonstrated in 2004 by Lynch et al. (12) and Paez et al. (13) who demonstrated that patients with EGFR mutations present in the tumors of patients with non-small cell lung cancer exhibited a dramatic response to getfitinib, the epidermal growth factor (EGFR) tyrosine kinase inhibitor (TKI), bringing personalized medicine to reality for a subset of NSCLC patients (12, 13).

Utilization of expensive systemic targeted therapies, however, has traditionally required invasive biopsies in order to assess for targetable tumoral aberrations. This presents a challenge for the monitoring of lung cancers due to the requirement for longitudinal sampling of tumors (14).

## Metastasis and epithelial-mesenchymal transition

Metastasis is an extremely complex, multistep process. Cells must gain the ability to intravasate into the blood from the bulk tumor, travel through the blood undergoing sheer stressors and immune evasion, and extravasate to favorable metastatic sites such as bone, brain and liver (15–17). In order to detach from the primary tumor and disseminate into the blood, cells must undergo a cellular process known as epithelial-mesenchymal transition (EMT) (18). EMT enables tumor cells to become motile and enhances migratory capabilities which in effect allows cells to penetrate into the lymph vasculature and circulate as single or clusters of circulating tumor cells (CTCs) (19). Whilst in blood, CTCs exist in a dynamic EMT state (20). CTCs extravasate having undergone the reverse process known as mesenchymal to epithelial transition (MET) and colonize at distant organs, (21). EMT is thought to support cell invasiveness but restrict proliferation, thereby maintaining cancer cell survival in metastatic sites whereas MET re-activates proliferative potential (22). The famous “seed and soil” hypothesis proposed by Stephen Pagent in the Nineteenth century suggesting that tumor cells (the “seed”) have a preference to metastasize in certain organs (the ‘soil) (23). This hypothesis has since been revisited by Fidler and Langly, still holding significance in cancer research today (24, 25).

## Circulating tumor cells in lung cancer

CTCs were first described by an Australian physician, Thomas Ashworth in 1869, where cancer cells in the blood were observed which resembled the cells of the primary tumor (26). CTCs play a central role in the metastatic spread of lung cancer, that is ultimately responsible for patient morbidity and mortality from the disease (27). While the concept of CTCs were described over one hundred years ago, it is only recently that they have been utilized in cancer diagnosis and prognosis (28).

Evidence has shown that the presence of CTCs in the blood correlates with poor overall survival in patients with metastatic prostate, breast and colon cancers (29–31). Patients with SCLC have on average 10 times more CTCs than patients with any other tumor type (32–34).

Molecular targeted therapies such as tyrosine kinase inhibitors (TKIs) in epidermal growth factor receptor (EGFR) mutants and anaplastic lymphoma kinase (ALK) inhibitors in ALK rearranged NSCLC patients have recently advanced the management of lung cancer for a limited proportion of patients (35–39). To determine eligibility for such targeted therapies, tumor biopsies have traditionally been necessary, increasing the likelihood of biopsy-related complications (40). Even in patients developing resistance to first line EGFR TKIs, liquid biopsies using circulating tumor DNA plasma only detect T790M mutations in ~80% of cases, particularly in low volume disease, making a repeat biopsy necessary. Tumor heterogeneity within the primary site or between primary and metastatic sites, can also create potential sampling bias, which may mask the true genetic profile of the cancer. The prospect of longitudinal sampling in order to monitor for the development of therapeutic resistance to treatments is likewise limited if invasive biopsies are essential (41, 42).

Use of CTCs as a liquid biopsy is promising for serial assessment of tumor evolution during the course of the disease and during systemic treatment in a less invasive, real-time manner, by a simple blood draw (19, 43). This liquid biopsy also provides potential for the early diagnosis of cancer and valuable insights into tumor heterogeneity and genomic diversity for the early diagnosis of cancer and guidance of clinical treatment (44, 45). A sensitive and unbiased isolation method to capture CTCs is therefore essential to provide tumoral material for analysis and potentially drive treatment decisions (46, 47).

## Circulating tumor cell detection methods in lung cancer

CTCs have the potential to accompany standard screening tests and be used for molecular characterization of a tumor (48). Detection of CTCs in NSCLC has been challenging due to the rarity in circulation (a few CTCs per billion normal blood cells) and the presence of non-epithelial characteristics (49). It is therefore imperative that sensitive and specific CTC detection methods are developed and optimized to assist in better patient monitoring and management (50–54). The advantages and disadvantages of the isolation methods in lung cancer are discussed and summarized in the Table 2. A summary of the CTC lung cancer studies are highlighted in Table 3.

**Table 2:** The Summary of different Circulating Tumor Cell isolation methods currently used in research.

**Isolation method**	**Mode of action**	**Advantages**	**Disadvantages**	**Examples**
FDA approved (clinical trials)	EpCAM positive based selection	Has become the “gold standard” for validation of CTCs with an epithelial phenotype. High reproducibility. High specificity. FDA-approved method.	CTCs can undergo EMT which may result in reduced expression of epithelial markers, leading to loss of effective capturing of cells with mesenchymal characteristics following EMT.	Cellsearch (Menarini Silicon Biosystems, Italy)
Positive Immunoselection	EpCAM positive based selection	Ability to process larger volumes of blood for the capture of higher numbers of CTCs.	As above	GILUPI CellCollector (GILUPI Nanomedizin) (55) Ephesia CTC-chip (56)
Negative Immunoselection	Depletion of Leukocytes by CD45 Antibodies	Has the ability to avoid false-negative results or loss of CTCs due to phenotypic heterogeneity.	CTCs are often contaminated with remaining blood cells resulting in low purity.	RosetteSep (StemCell Technologies, Canada) (57) EPISPOT (Epithelial Immunospot Assay; France) (58)
Size-Based Filtration	Cells are separated using filtration to remove smaller cells in the blood (e.g., White blood cells)	Simple process.	Will exclude small sized CTCs, filter clogging and limited blood processing/filter are potential problems.	Screen Cell (France) MetaCell (Ostrava, Czech Republic) Isolation by Size of Epithelial Tumor cells (ISET) (Rarecells Diagnostics, Paris, France) (59) Microdevice- Cote's group (60) Parsortix (61) Microcavity array System (MCA) (62)
Density-based Filtration	Cells are separated based on different densities after centrifugation.	Cells separated into distinct layers	CTC size and density not uniform CTCs may get lost in plasma or by formation of CTC aggregates Poor sensitivity	Ficoll Lymphoprep (Stem Cell Technologies, Vancouver, Canada) (63) OncoQuick (VWR, Radnor, PA) Accucyte (64)
Microfluidics	Cells are separated based on their biological or physical properties	Higher sensitivity, purity, lower cost, reduced sample size, short processing time, compatibility with downstream assays	Small CTCs of comparable size to WBCs would typically be missed Cell morphology may be altered due to high shear stress during microfiltration	Isoflux (Fluxion Biosciences) (65) CTC iChip (Nagrath) (66, 67) ClearCell FX/Spiral Microfluidics (ClearbridgeBiomedics, Singapore) (54) Herringbone Chip (Nagrath) (31)
Immunomagnetic	Enriches target cells and eliminates cells that are not bound to magnetic particles	Isolate cells easily accessible	Nonspecific contamination can be from adsorption of background cells to the capturing device	MagSweeper (Jeffrey Lab, Stanford, CA) (68) AdnaTest (Qiagen, Hannover, Germany) (69) Magnetic Activated Cell Sorting System (Miltenyi Biotec, Germany) (70, 71) MagSifter (72)
Electrophoresis	Cells are separated based on their electrical signature using an electric field	Single-cell-level precision High accuracy and precision	Process can be slow resulting in low sample throughput	DEPArray (Silicon Biosystems)
Enrichment free platforms	Cells are detected through imaging platforms with no need for enrichment due to advancements in fluorescence imaging	Multiple analysis parameters can be used to identify and characterize specific populations of interest High specificity and sensitivity. No need for enrichment.	Potential for high speed imaging to reduce resolution thereby worsening accuracy.	HD-CTC (EPIC Sciences, California) (44, 73, 74) FastCell (SRI Biosciences) (75) CytoTrack (Denmark) (76, 77)

**Table 3:** Summary of a number of Circulating Tumor Cell studies in lung cancer.

**Study**	**Histology**	**Sample number**	**Isolation method**	**Major findings**	**References**
Das et al., 2012	NSCLC	57	FastCell	CTCs were detected in 42% of patients.	(78)
Devriese et al., 2012	NSCLC	46	Cellsearch	CTCs were detected in 62% of patients. 30% of patients positive for CK7 and 9% positive for CK19.	(79)
Hiltermann et al., 2012	SCLC	59	Cellsearch	Lower number of CTCs in patients with early stage SCLC. CTC decrease after one cycle of chemotherapy- no change after four cycles	(80)
Hirose et al., 2012	NSCLC	33	Cellsearch	CTCs were detected in 36.4% of patients and 15.2% had five or more CTCs before chemotherapy. No difference in response to chemotherapy between CTC-positive and CTC-negative patients. Progressive disease higher in CTC-positive patients.	(81)
Hofman et al., 2012	NSCLC	250	ISET	CNHC's were detected in 49% of patients corresponding to malignant (41%), uncertain malignant (6% and benign cells (2%) respectively.	(82)
Hou et al. 2012	SCLC	97	Cellsearch	CTCs present in 85% of patients. OS of 5.4mths for ≥50 CTCs/7.5ml blood	(83)
Illie et al., 2012	NSCLC	87	ISET	CTCs positive for *ALK* from 5 patients corresponded to patients having *ALK*-rearrangement in tumors.	(84)
Isobe et al., 2012	NSCLC	24	Cellsearch	CTCs detected in 33.3% of patients	(85)
Krebs et al., 2012	NSCLC	45	Cellsearch/ISET	CTCs detected in 80% of patients using ISET compared to 23% of patients using Cellsearch. Subpopulation of cells detected by ISET did not express epithelial markers	(86)
Naito et al., 2012	SCLC	51	Cellsearch	Patients with ≥8 had worse survival than those with < 8 CTCs.	(87)
Punnoose et al., 2012	NSCLC	41	Cellsearch	CTCs were detected in 78% of patients at baseline. High baseline CTC counts associated with response to treatment. Decreased CTCs associated with PFS.	(88)
Saucedo-Zeni et al., 2012	NSCLC	24	GILUPI CellCollector	CTCs were successfully enriched from over 90% of patients with breast cancer or non-small cell lung cancer.	(55)
Wendel et al., 2012	NSCLC	78	HD-CTC assay	CTCs were detected in 73% of patients. No significant difference between stages.	(89)
Funaki et al., 2013	NSCLC	130	Rosette Sep	ITCs were detected in 74% of patients.	(90)
Hosokawa et al., 2013	NSCLC	22	MCA	CTCs were detected in 77% of patients using the MCA system versus 32% using the Cellsearch system. MCA system also isolated CTC clusters from patients identified as CTC-negative using Cellsearch.	(91)
Ni et al., 2013	NSCLC and SCLC	11	Cellsearch	Copy number variations reported from single CTCs similar to that of the metastatic tumor of the same patient.	(92)
Pailler et al., 2013	NSCLC	18	Cellsearch/ISET	ALK rearrangements detected in CTCs of patients with ALK positive NSCLC enabling monitoring and testing of crizotinib.	(93)
Swennenhuis et al., 2013	NSCLC and SCLC	10	Cellsearch	CTCs from 25% of patients were identified and single CTCs were isolated and amplified.	(94)
Carlsson et al., 2014	NSCLC	129	HD-CTC assay	Presence of CTM combined with clinical and imaging data assisted in discriminating for diagnostic accuracy in all NSCLC patients.	(95)
Earhart et al., 2014	NSCLC	6	Magnetic Sifter	CTCs detected in 100% of patients.	(96)
Illie et al., 2014		168	ISET	CTCs were detected in 3% of COPD patients	(97)
Juan et al., 2014	NSCLC	37	Cellsearch/ISET	ALK rearrangements detected in CTCs of patients with ALK positive NSCLC enabling monitoring and testing of crizotinib.	(98)
Marchetti et al., 2014	NSCLC	37	Cellsearch	CTCs were detected in 41% of patients. EGFR mutations identified by NGS in 84% of patients.	(99)
Muinelo –Romay et al., 2014	NSCLC	43	Cellsearch	At baseline 41.9% of patients were positive for CTCs. Patients with ≥5 baseline had worse PFS and OS. Patients with increased levels of CTCs has worse PFS and OS.	(100)
Nel et al., 2014	NSCLC	43	Negative depletion	Increased CD133-positive to pan-CK-positive cell type ratio (stem like to epithelial ratio) and presence of mesenchymal N-cad-positive cells, associated with shorter PFS.	(101)
Normanno et al., 2014	SCLC	60	Cellsearch	At baseline 90% of patients were positive for CTCs and strongly associated with organs involved. CTC reduction as high as 89% following chemotherapy.	(102)
Chudsama et al., 2015	NSCLC	20	Screen Cell	An increase in CTCs following EC observed in 75% of patients. Could have implications for tumor dissemination and metastatic spread.	(103)
Dorsey et al., 2015	NSCLC	23	Density gradient centrifugation	CTCs positive in 65% of patients. CTC count reflect clinical course and response to treatment.	(104)
Tu et al., 2015	NSCLC and SCLC	18	Cellsearch	CSFTC were positive in 78% of MRI confirmed LM samples. CSFTC clusters were observed in 67% of patients.	(105)
Aieta et al., 2016	NSCLC	1	Cellsearch	Presense of EML4-ALK+ CTCs at baseline. EML4-ALK+ CTCs could be interpreted as resistance sign to crizotinib treatment leading to progressive disease.	(106)
Cheng et al., 2016	SCLC	89	Cellsearch	CTCs positive in 87.6% of patients. CTC count independent indicator for PFS and OS.	(107)
Crosbie et al., 2016	NSCLC	27	Cellsearch	CTCs positive in 22% of patients at baseline. CTC detection at baseline associated with reduced DFS and 3-year survival.	(108)
Hanssen et al., 2016	NSCLC	48	Cellsearch	CTCs positive in 15% of patients. CTC positivity was associated with patient disease state.	(109)
He et al., 2016	NSCLC	66	Cellsearch	Presence of CTCs at baseline associated with significantly shorter PFS.	(110)
Morrow et al., 2016	NSCLC	1	Cellsearch	CDX derived from CTCs enriched from NSCLC patient.	(111)
Nicolazzo et al., 2016	NSCLC	24	Cellsearch	Patients with PD-L1 negative CTCs all had clinical benefit, while patients with PD-L1 (+) CTCs all experienced progressive disease.	(112)
Tan et al., 2016	NSCLC	27	ClearCell FX	CTCs positive in 100% of patients, 14 were *ALK*-positive.	(113)
Zhang et al., 2016	NSCLC	46	Negative immunoselection	CTCs positive in 87% of patients. CTC count of more than eight prior to chemotherapy was a strong predictor of PFS.	(114)
Chudsama et al., 2017	NSCLC	10	ScreenCell	A significant increase in CTCs was observed from baseline levels following lung manipulation.	(115)
Chudsama et al., 2017	NSCLC	23	ScreenCell	CTCs positive in 78.3% and 73.9% reviewed by 2 pathologists.	(116)
Coco et al., 2017	NSCLC	73	ScreenCell	Baseline CTC count had no significant association with OS or PFS.	(117)
Illie et al., 2017	NSCLC		Cellsearch/ISET	CTCs positive in 32% of patients evaluated on Cellsearch. CTCs positive in 75% of patients evaluated on ISET. Expression of MET was positive in 72% of cases.	(118)
Lindsay et al., 2017	NSCLC	125	Cellsearch	CTCs positive in 40.8% of patients. Patients with ≥2 CTCs at baseline had poorer prognosis.	(119)
Messaritakis et al., 2017	SCLC	64	Cellsearch	CTCs positive in 50% of patients before treatment. Pazopanib treatment significantly reduced proportion of patients with increased CTC numbers. High CTC number at baseline correlated with reduced PFS and OS. Detection of VEGFR2^+^ CTCs during treatment could be associated with resistance to pazopanib.	(120)
Messaritakis et al., 2017	SCLC	108	Cellsearch	CTCs positive in 60.2% of patients at baseline. Presence of proliferative (CK67+) and non-proliferative (Ki67-), apoptotic (M30+) and non-apoptotic (M30-) as well as EMT (Vim+) CTCs were present in the same patient.	(121)
Pailler et al., 2017	NSCLC	39	Cellsearch/ISET	Significant association between the decrease in CTC number with *ALK*-CNG on crizotinib and longer PFS. *ALK*-CNG may be a predictive biomarker for crizotinib efficacy in *ALK*-rearranged NSCLC patients.	(122)
Salgia et al., 2017	SCLC	42	Cellsearch	CTCs positive in 83% of patients at baseline. Presence of CTCs at baseline were prognostic of shorter PFS and OS.	(123)
Tong et al., 2017	NSCLC	127	Negative immunoselection	CTCs positive in 80.31% of patients at baseline. Patients with post-treatment increases in CTC count had poorer OS and PFS than those without increases. Baseline CTC count and change in CTC count during treatment were valuable prognostic indicators for NSCLC.	(124)
Wang et al., 2017	SCLC	42	Negative immunomagnetic enrichment	CTCs positive in 76.19% of patients with SCLC and negative in controls. PFS correlates with CTC numbers and the change in CTC numbers after 1 cycle of chemotherapy.	(125)
Yang et al., 2017	NSCLC	107	Cellsearch	CTCs positive in 44% of patients at baseline. CTC >5 at baseline was a strong negative predictor of PFS and TTF. Five or more CTCs on day 28 were strongly associated with a poor PFS.	(126)
Yuanling et al., 2017	NSCLC	105	Cellsearch	CTCs positive (≥2) in 29% of patients at baseline and 9% had ≥5 CTCs. CTC count of ≥5 CTCs correlated with poor PFS and OS.	(127)
Alamgeer et al., 2018	SCLC	28	Cellsearch	At baseline, two or more CTCs were detected in 86.6% of patients.	(128)
Guibert et al., 2018	NSCLC	96	ISET	CTCs positive in 93% of patients at baseline. CTCs more frequently PD-L1^+^ than tissue (83 vs. 41%). Pre-treatment high CTC counts associated with increased risk of death and progression. Pre-treatment PD-L1^+^CTCs associated with bad prognosis in patients treated with PD-1 inhibitors.	(129)
Milano et al., 2018	NSCLC	10	Density gradient centrifugation	CTCs undergoing EMT (CTCs^EMT^) positive in 30% of patients. CTCs^EMT^ detection related to poor therapeutic response.	(130)
Tong et al., 2018	NSCLC	43	Negative immunoselection	CTCs positive in 76.7% of patients at baseline. CTC count was a strong predictor of PFS and OS.	(131)

*NSCLC, non-small cell lung cancer; SCLC, small cell lung cancer; CTC, circulating tumor cells; CNHCs, circulating non-hematological cells; ITC, isolated tumor cells; CTM, circulating tumor microemboli; OS, overall survival; PFS, progression-free survival; TTF, time-to-treatment failure; COPD, chronic obstructive pulmonary disease; EC, endobronchial cryotherapy; CSFTC, cerebrospinal fluid tumor cell; MRI, Magnetic Resonance Imaging; LM, leptomeningeal metastasis; NGS, next-generation sequencing; CDX, cell line-derived xenograft*.

## *Ex-vivo* expansion of circulating tumor cells

Despite limitations of current CTC isolation techniques, these cells have been detected in a number of cancers, including breast, head, and neck cancer, lung, prostate, colon and gastric cancer (21, 50, 53, 109, 132, 133–135). Successful *ex-vivo* culture of CTCs represents a “Holy Grail” in the study of cancer metastasis as it allows for in depth characterization of metastasis initiating cells as well as the testing of functional assays (136).

**Figure 1:**
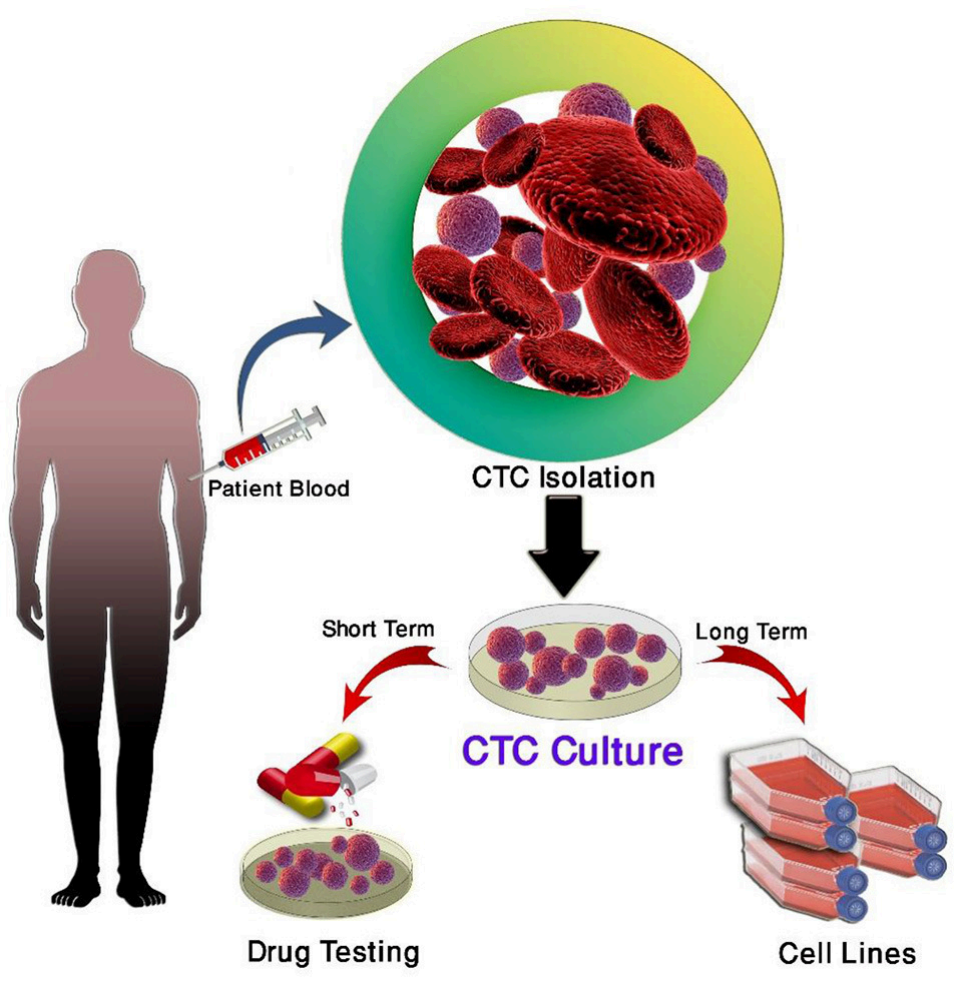
Culture of circulating tumor cells.

Short-term CTC culture (3–14 days) has been achieved in a number of cancer types, even from early stage cancers (137–139). This allows for the recapitulation of the disease in an *ex vivo/in vivo* setting for the testing of therapies and functional analysis (140). A summary of this is in Table 4. In comparison, long-term cultures have only been established in advanced metastatic cases where a large number of CTCs have been isolated (111, 142, 143) (Table 5). Long-term culture studies have shown that some CTCs in patient blood are immortalized and can be cultured *ex vivo* into stable cell lines (Figure 1) (139). There are only a few reports of successful long-term culture, notably, in patients with advanced stages of disease (136, 145, 146). CTC-expansion has been limited due to the influence of CTC enrichment. Certain cancers also require specific culture conditions for primary and metastatic samples (136). The successful culture of CTCs long-term holds great promise in developing personalized cancer treatment for testing of therapeutic efficacy using drug screening (140). This approach could assist in determining the choice of therapeutic regimen beneficial for patients and hence holds significance in advancement of precision medicine and personalized oncology (139).

**Table 4:** Summary of short-term Circulating Tumor Cell culture in Lung cancer.

**Study**	**Method of CTC isolation**	**CTC culture conditions**	**Group size**	**Morphology**	**Reference**
Zhang et al., 2014	*In situ* capture and culture	4 Culture Conditions: 1. 3D co: Collagen, matrigel and cancer associated fibroblasts (from pancreatic tumor) 2. 3D mono: Only gel culture 3. 2D co: Only cancer associated fibroblasts 4. 2D mono: No gel or fibroblasts	14	Spheroids	(141)

**Table 5:** Summary of long-term Circulating Tumor Cell culture in Lung cancer.

**Study**	**Histology**	**Method of CTC isolation**	**CTC culture conditions**	**Group size**	**CTC lines established**	**Morphology/Histology**	**References**
Hodgkinson et al., 2014	SCLC	RosetteSep/ Ficoll/ xenotransplantation	Xenotransplantation	6		Morphology of CDX macrometastases: 1. Clusters 2. Sheets of densely packed small round or oval cells 3. Scant cytoplasm 4. Enlarged/inconspicuous nuclei 5. Speckled chromatin 6. Focal nuclear molding	(142)
Hamilton et al., 2015	SCLC	Ficoll-Hypaque	RPMI 1640 medium, serum-free (insulin, IGF-1, selenite)	30	3	Spheroids or attached	(143)
Morrow et al., 2016	NSCLC	RosetteSep/Ficoll/ xenotransplantation	Xenotransplantation	1		Morphology of CDX macrometastases: 1. Diffuse sheets of large polygonal cells 2. Abundant eosinophilic cytoplasm 3. Vesicular chromatin 4. Enlarged nucleoli	(111)
Drapkin et al., 2018	SCLC	RosetteSep/Ficoll/ xenotransplantation	Xenotransplantation	46		Cytoplasmic expressions of chromogranin, synaptophysin and/or CD56 as well as the lack of CD45 expression confirmed diagnosis	(144)

Three main strategies are used for the propagation of CTCs in culture; two-dimensional (2D) culture, very commonly used for expansion of CTCs short-term, three-dimensional (3D) culture used for long-term expansion and xenotransplantation and four dimensional (4D) shown to mimic the process of metastasis (137, 147–150).

The expansion of CTCs *in-vivo* to generate patient derived xenografts (PDXs) may also be used to comprehensively analyse advanced disease biology and present a valuable model to understand cancer metastasis. The use of PDX's have been shown to mimic patient's disease and mirror response to chemotherapy (e.g., Platinum agents) (142, 151). However, PDXs have been challenging due to CTC heterogeneity causing unreliability of these models to translate clinically. PDX model development also takes 4–8 months and therefore are not optimal for rapid studies necessary for patients with advanced disease (151). In an ideal world cancer cell lines would be routinely generated from each cancer patient but this is not realistic at present (136, 139, 152).

## Clinical significance

The immediate need for early detection of lung cancer recurrence and monitoring treatment response is essential to facilitate improved survival of patients. Previous studies have shown computerized tomography (CT) screening has helped to reduce mortality, however CT has risks such as radiation exposure, leading to an increased risk of long-term cancer (153). This signifies the need for less invasive techniques for the early detection of metastasis and aid the personalized treatment of lung cancer. The use of CTCs as a liquid biopsy has the potential to accompany standard screening tests and also allow for molecular and genetic characterization of the tumor (48).

Enumeration of CTCs could provide a biomarker for cancer surveillance following treatment of early, locally advanced and advanced lung cancer and provided a better understanding on the mechanisms of metastasis (33). Although chemotherapy, targeted small molecules and immune checkpoint inhibitor therapies have shown significant benefits, the occurrence of acquired drug resistance and disease relapse are very common. Through serial sampling a longitudinal analysis of CTCs for identification of tumor evolution could provide valuable insights into mechanisms underlying resistance (154).

Detection of CTCs in lung cancer has been challenging, as CTCs usually present with non-epithelial characteristics (49). This emphasizes the need for more sensitive technologies to better capture CTCs for in-depth characterization and functional studies using cell culture and xenograft models. This will then ultimately assist in optimizing personalized therapies for lung cancer patients, with CTCs potentially being a prognostic biomarker.

## Conclusion

The clinical significance of CTCs is yet to be established, however, advances in CTC detection and single-cell profiling have significantly improved our knowledge of underlying mechanisms of the evolution and dissemination of cancer and is progressively being translated to clinical studies. With lung cancer being the largest cause of cancer mortality worldwide, one of the biggest challenges for managing and treating patients is the lack of early screening/diagnostic methods (4). The isolation of CTCs from cerebrospinal fluid (CSF), may represent a unique subpopulation CTCs with ability to survive the journey in blood circulation and subsequent invasion of the CNS (105, 155). CTCs hold great promise as biomarkers for the early diagnosis and treatment selection of patients as well as broadening the current knowledge of metastasis (154).

Recurrence and progress of the disease, severity of symptoms and side-effects dramatically decrease patient's quality of life (QoL) (156). Therefore there is a vital need to monitor tumor evolution and understand mechanisms underlying development of therapeutic resistance.

Challenges for the field to address include the low sensitivity and specificity of current technologies prohibiting their use in current clinical settings, the large number of CTCs required for the development of CTC lines and patient xenografts for downstream functional analyses and the limited number of CTCs frequently found in patients with early stage disease (157). CTCs have demonstrated prognostic clinical utility is breast, lung and prostate cancers using the CellSearch technology (158, 159). Recent studies have demonstrated renewed interest in the FDA-approved Cellsearch platform for CTC PD-L1 analysis (160–162). These studies demonstrate how CTCs could be used to identify patients for anti PD-1/PD-L1 therapy (immunotherapy). Cellsearch relies on CTC enrichment using EpCAM (when CTCs undergo EMT, EpCAM is downregulated). As such the field is moving toward label-free technologies for CTC isolation. Currently, there are a number of technologies to enrich CTCs (i.e., Rarecyte, iChip, ISET, DEPArray, EPISPOT etc). The current label-free technologies are being validated for a number of cancers in larger clinical trials (163, 164). This is highlighted by the Cancer-ID network consortium in standardizing CTC/ctDNA and exosome isolation, analysis and reporting (165). The current gold standard in isolating CTCs from patient blood relies on the EpCAM status of these cells, thereby excluding a large majority of CTCs present in the blood of metastatic patients. Furthermore, Cellsearch does not allow for subsequent culture as the cells are fixed (166). CTCs as a liquid biopsy have valuable potential to improve early diagnosis, monitoring of disease, and direct treatment of lung cancer, however a better understanding of CTC biology is crucial for the field to move forward.

## Author contributions

JK, AK, KO, CP: Idea. JK, AK, MW: Preparation of figures and tables. All authors were involved in the preparation, review and editing of the manuscript.

### Conflict of interest statement

The authors declare that the research was conducted in the absence of any commercial or financial relationships that could be construed as a potential conflict of interest.
